# A time-series meta-transcriptomic analysis reveals the seasonal, host, and gender structure of mosquito viromes

**DOI:** 10.1093/ve/veac006

**Published:** 2022-02-02

**Authors:** Yun Feng, Qin-yu Gou, Wei-hong Yang, Wei-chen Wu, Juan Wang, Edward C Holmes, Guodong Liang, Mang Shi

**Affiliations:** Department of Viral and Rickettsial Disease Control, Yunnan Provincial Key Laboratory for Zoonosis Control and Prevention, Yunnan Institute of Endemic Disease Control and Prevention, No. 5 Wenhua Road, Xiaguan, Dali, Yunnan 671000, China; Shenzhen Campus of Sun-Yat Sen University, Sun-Yat Sen University Shenzhen Campus, Guangming New District, Shenzhen, Guangdong 518107, China; Department of Viral and Rickettsial Disease Control, Yunnan Provincial Key Laboratory for Zoonosis Control and Prevention, Yunnan Institute of Endemic Disease Control and Prevention, No. 5 Wenhua Road, Xiaguan, Dali, Yunnan 671000, China; Shenzhen Campus of Sun-Yat Sen University, Sun-Yat Sen University Shenzhen Campus, Guangming New District, Shenzhen, Guangdong 518107, China; Department of Viral and Rickettsial Disease Control, Yunnan Provincial Key Laboratory for Zoonosis Control and Prevention, Yunnan Institute of Endemic Disease Control and Prevention, No. 5 Wenhua Road, Xiaguan, Dali, Yunnan 671000, China; Sydney Institute for Infectious Diseases, School of Life and Environmental Sciences and School of Medical Sciences, The University of Sydney, Sydney, NSW 2006, Australia; State Key Laboratory of Infectious Disease Prevention and Control, National Institute for Viral Disease Control and Prevention, Chinese Center for Disease Control and Prevention, 155 Changbai Road, Changping District, Beijing 102206, China; Shenzhen Campus of Sun-Yat Sen University, Sun-Yat Sen University Shenzhen Campus, Guangming New District, Shenzhen, Guangdong 518107, China

**Keywords:** mosquito, virome, virome ecology, virus discovery, time-series, meta-transcriptomics

## Abstract

Although metagenomic sequencing has revealed high numbers of viruses in mosquitoes sampled globally, our understanding of how their diversity and abundance varies in time and space as well as by host species and gender remains unclear. To address this, we collected 23,109 mosquitoes over the course of 12 months from a bat-dwelling cave and a nearby village in Yunnan province, China. These samples were organized by mosquito species, mosquito gender, and sampling time for meta-transcriptomic sequencing. A total of 162 eukaryotic virus species were identified, of which 101 were novel, including representatives of seventeen RNA virus multi-family supergroups and four species of DNA virus from the families *Parvoviridae, Circoviridae*, and *Nudiviridae*. In addition, two known vector-borne viruses—Japanese encephalitis virus and Banna virus—were found. Analyses of the entire virome revealed strikingly different viral compositions and abundance levels in warmer compared to colder months, a strong host structure at the level of mosquito species, and no substantial differences between those viruses harbored by male and female mosquitoes. At the scale of individual viruses, some were found to be ubiquitous throughout the year and across four mosquito species, while most of the other viruses were season and/or host specific. Collectively, this study reveals the diversity, dynamics, and evolution of the mosquito virome at a single location and sheds new lights on the ecology of these important vector animals.

## 1. Introduction

The development of metagenomic next-generation sequencing (mNGS) has led to explosion of virus identification in arthropods, with more than 2,000 novel virus species documented over the past 5 years (Li et al. 2015a; Shi et al. 2016; Wu et al. 2020). Among these arthropods, mosquitoes (class Insecta, family Culicidae) are subject to intensive virome investigation because they act as the major vectors of human and animal pathogens such as the dengue, yellow fever, and Zika viruses (Favia 2015; Wu et al. 2020). More than fifty-six mosquito-associated viruses have been identified in China alone (Atoni et al. 2020), although those relevant for mammalian/human infection—the so-called arthropod-borne viruses (arboviruses) or vector-borne viruses—comprise only a very small proportion (Li et al. 2015a; Shi et al. 2017). Most of the expansion has occurred in those mosquito viruses that do not infect vertebrates and hence are classed as arthropod-specific (Shi et al. 2016; Xia et al. 2018a).

While the diversity of the mosquito virome continues to expand, how their genetic structure and ecological dynamics change by host species, through time and space, and by mosquito gender remains largely unclear, with detailed epidemiological investigations limited to individual vector-borne viruses (e.g. dengue (Tian et al. 2016), yellow fever (Cunha et al. 2020), and Zika viruses (Wang et al. 2021)) and some arthropod-specific viruses (e.g. Wutai mosquito phasivirus (Ribeiro et al. 2019) and Culex flavivirus (Moraes et al. 2019)). However, we lack a characterization at the scale of entire viromes. Importantly, total RNA sequencing (meta-transcriptomics) enables characterization of the full range of microbes (i.e. the total ‘infectome’) within species and also provides an estimation of their relative abundance, thereby helping to transform studies of viral ecology (Shi, Zhang, and Holmes 2018, Shi et al. 2018). Recent metagenomic studies in Australia (Shi et al. 2017), China (Xia et al. 2018a), Europe (Pettersson et al. 2019), Guadeloupe (Shi et al. 2019), and the USA (Batson et al. 2021) have demonstrated that virome composition and diversity are generally structured by mosquito taxonomy, suggesting that the host species barrier is an important factor in shaping patterns of interspecies virus transmission. To date, however, few studies have investigated how mosquito viromes change through time within a year, even though there have been suggestions that some arthropod-specific viruses may be effectively endemic (Lee et al. 2019). Revealing the dynamics of mosquito virome diversity and abundance in time requires multiple longitudinal samples taken throughout the course of a year, simultaneously controlling for factors such as geographic location, mosquito species, and gender.

Yunnan province is located in southwest China, sharing borders with Myanmar, Vietnam, and Laos (Wu et al. 2017), and is adjacent to Tibet, Sichuan, and Guangxi provinces (Sun et al. 2009). It is characterized by a tropical to subtropical climate, contains a number of biodiversity hotspots (Cao et al. 2011; Feng et al. 2012; Zuo et al. 2014), and is notorious for harboring viruses that have the potential to result in human infection (Hu et al. 2017a; Yang et al. 2019), including mosquito-borne diseases (Zhang et al. 2014; Wu et al. 2017; Mao and Zhou 2020; Zhao et al. 2020). More than ten arboviruses have been isolated in Yunnan province, including Japanese encephalitis virus (JEV) (Liu et al. 2013), dengue virus (Hu et al. 2017a; Hu et al. 2017b), chikungunya virus (Yin et al. 2021), Zika virus (Zhu et al. 2020), and Banna virus (BAV) (Wang et al. 2015), making Yunnan one of the most important places in China for mosquito control (Xia et al. 2018b). In addition, mNGS has identified vector-borne viruses such as Mammalian rubulavirus 5 and Getah virus (Liu et al. 2021). It is therefore of great importance to understand the diversity and ecological dynamics of mosquito-associated viruses in Yunnan.

 To understand the factors that shape mosquito viromes, we conducted a time-series meta-transcriptomic analysis of 23,109 mosquitoes collected at a single location in Yunnan province, comparing the diversity and abundance of all the RNA and DNA viruses that could be detected through time.

## 2. Materials and methods

### 2.1 Mosquito sampling and sample processing

From January to December in 2018 we performed year-round sampling at two locations in Xiangyun county, Yunnan province, China: (i) Qinghua cave, a bat-dwelling cave, and (ii) Shuizhangdi, a nearby village located 5 km from the cave. Mosquitoes were trapped using an ultraviolet device without carbon dioxide supplementation at each location, and those with obvious signs of blood-feeding were intentionally removed. Precise sampling location and time were recorded. Mosquito gender was assessed by antennae patterns (Sallum et al. 2020). Species identifications were initially carried out based on morphological characteristics by experienced field biologists and further confirmed by analyzing the cytochrome C oxidase subunit I (COI) gene (Delgado-Serra et al. 2021) from the sequencing results. The samples were then transported by liquid nitrogen to the laboratory and transferred to −80°C storage freezer.

### 2.2 RNA library construction and sequencing

Samples were organized into forty-eight pools based on time, host, and gender and homogenized with a TissueLyser in 600 µl of lysis buffer. Total RNA extraction and purification were performed using the RNeasy Plus Mini Kit (QIAGEN), following the manufacturer’s instructions. An Agilent 2100 Bioanalyzer was used to evaluate the quality of the extracted RNA. Total RNA sequencing libraries were constructed after removing host rRNA using the Ribo-Zero-Gold (Human–Mouse–Rat) Kit (Illumina). Paired-end (100 bp) sequencing of the forty-eight dual-indexed libraries was then performed on an Illumina HiSeq 4000 platform. All library construction and sequencing were performed by Novogene (Beijing, China). All sequencing reads have been deposited in the Sequence Read Archive (SRA) database under the BioProject accession PRJNA778885.

### 2.3 Virus discovery and characterization

Adaptor sequences and low-quality reads were first removed from the raw sequencing reads. The quality-controlled reads were then assembled *de novo* into contigs using Megahit version 1.2.8 (Li et al. 2015a; Li et al. 2015b). The assembled contigs were subsequently compared against the nonredundant protein (nr) database downloaded from GenBank using Diamond blastx version 0.9.25 (Buchfink, Xie, and Huson 2015), with an *e*‐value threshold of 1E − 5 to retain high sensitivity at a low false-positive rate. Viral contigs were first identified based on the taxonomic information of each blast hit, and potential false positives were removed by blasting against the nonredundant nucleotide database (nt). The remaining viral contigs were merged to form longer genomes. To eliminate mis-assembly, reads were mapped back to the virus genome using Bowtie2 version 2.3.5.1 (Deorowicz et al. 2019) and inspected using Geneious Prime version v.9.1.5 (Kearse et al. 2012).

To identify potentially novel viral species, we used a threshold of <90 per cent amino acid identity for the RdRp (RNA-dependent RNA polymerase) protein (RNA viruses), NS1 protein (parvoviruses), replicase protein (circoviruses), and DNA polymerase B protein (nudiviruses), in comparison with the closest related viruses. All viral genome sequences have been deposited in National Center for Biotechnology Information GenBank under accession numbers OL700045–OL700212.

### 2.4 Virus genome annotation and phylogenetic analysis

For each virus genome, open reading frames (ORFs) were predicted using TransDecoder version 5.5.0 (Onimaru et al. 2018). For annotation, these ORFs were compared against the Conserved Domain Database (Yang et al. 2020) as well as the nr database.

To infer the evolutionary history of the newly identified viruses, we performed interspecies phylogenetic analyses based on alignments of conserved proteins. Specifically, RdRp alignments were used for RNA viruses, while nonstructural protein 1 (NS1), replicase, and DNA polymerase alignments were used for DNA viruses. Reference viral genomes representative of background phylogenetic diversity were downloaded from the GenBank and aligned with viral sequences obtained from this study using MAFFT version 7 (Katoh and Standley 2013), with ambiguously aligned regions removed using TrimAL version 1.2rev59 (Capella-Gutiérrez, Silla-Martínez, and Gabaldón 2009). Phylogenetic trees were estimated using the maximum likelihood approach implemented in PhyML version 3.0 (Guindon et al. 2010), utilizing the LG + Γ model of amino acid substitution and the Subtree Pruning and Regrafting branch-swapping algorithm. The approximate likelihood ratio test with the Shimodaira–Hasegawa-like procedure was used to assess nodal support. In the case of JEV and BAV, intraspecific phylogenies were also estimated, based on the nucleotide genome sequences identified in this study as well as those from GenBank. Similar parameters were used as the phylogenetic analysis described above, with the exception that the General Time Reversible model was employed as the DNA substitution model.

### 2.5 Characterization of viral abundance and diversity

To determine virus abundance, we first removed reads associated with rRNA by mapping against the total rRNA database obtained from the SILVA website (https://www.arb-silva.de/). The remaining reads were mapped against the viral genome templates using Bowtie2 version 2.3.5.1 (Langmead and Salzberg 2012). The abundance for each virus species was subsequently estimated as the number of reads per million (RPM) using the formula ‘total mapped viral reads / total non-rRNA reads * 1,000,000’. To prevent false positives due to index hopping, a threshold of 0.1 per cent was applied for each virus species present in the same sequencing lane: that is, the libraries containing < 0.1 per cent of the most abundant library were treated as ‘negative’. For each library, alpha diversity, which reflects the observed richness (number of taxa) or evenness (the relative abundance of those taxa) of an average sample, was estimated using the Shannon diversity index (Lagkouvardos et al. 2017) at the virus species level using the *vegan* package (Komesu et al. 2020). The overall significance levels for each factor—i.e. time, host species, and gender—were evaluated using analysis of variance (ANOVA) and *t*-tests. Beta diversity (Loraine et al. 2015) (i.e. virus diversity between different libraries) was calculated using the Euclidean distance matrix and virome structure and plotted as a function of multidimensional scaling ordination (Alencar et al. 2018). A generalized linear model was used to investigate the effect of time, species, and gender on richness, abundance, and alpha diversity. All these statistical analyses were performed using R v 4.0.1 implemented in RStudio version 1.3.959, and graphs were plotted using the *pheatmap* (Ni et al. 2019) and *ggplot2* (Ito and Murphy 2013) packages implemented in R. The datasets and R code for the statistical tests and generalized linear model analysis are available at the figshare website under the link: https://doi.org/10.6084/m9.figshare.19064360.v1.

## 3. Results

### 3.1 Study design and sampling

**Figure 1.**
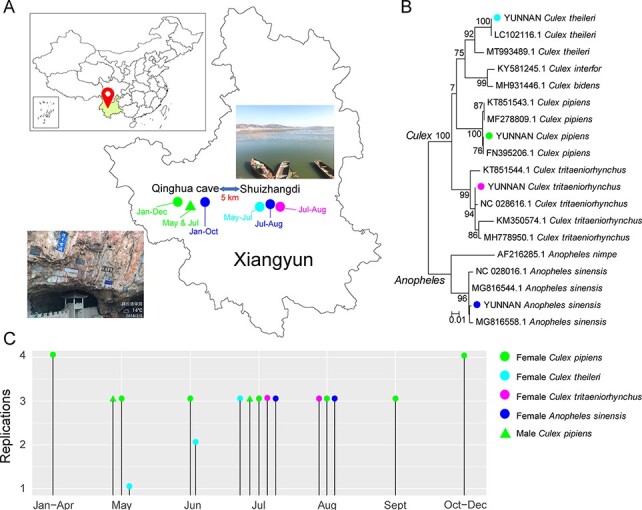
Organization of sampling and sequencing pools. (A) Locations and surrounding environments of Qinghua cave and Shuizhangdi village, Yunnan province, China, where the mosquito sampling was performed. (B) Mosquito species identification based on a maximum likelihood phylogeny of the COI gene revealed by meta-transcriptomic sequencing. (C) The experimental setting for this study: each line represents a group with a unique sampling location, time, and mosquito species, and the height of the line represents replications in that group. Throughout the figure, the color green represents *C. pipiens*, light blue denotes *C. theileri*, purple for *C. tritaeniorhynchus*, and deep blue for *A. sinensis*. Circles denote female mosquitoes while triangles denote males.

In 2018, we performed year-round sampling of mosquitoes in Xiangyun county, Yunnan province, China (Fig. 1A). A total of 23,109 mosquitoes were sampled, which contained four dominant species from two genera (*Culex* and *Anopheles*), among which *Culex pipiens* was mainly identified in Qinghua cave, while *Culex theileri, Culex tritaeniorhynchus*, and *Anopheles sinensis* were mainly identified in Shuizhangdi village (Fig. 1B). A representative proportion of the mosquitoes were subsequently divided into forty-eight pools, each comprising approximately fifty individuals, for meta-transcriptomic sequencing. These pools were further organized into sixteen groups based on mosquito species (*n* = 4), gender (*n* = 2, i.e. male versus female), and sampling time (*n* = 7, i.e. January–April, May, June, July, August, September, and October–December) (Fig. 1C). For most groups at least three pools were assigned as replicates (Fig. 1C). These groups formed the basis for later comparisons of the diversity and abundance of mosquito viromes.

### 3.2 Overview of the mosquito virome

**Figure 2.**
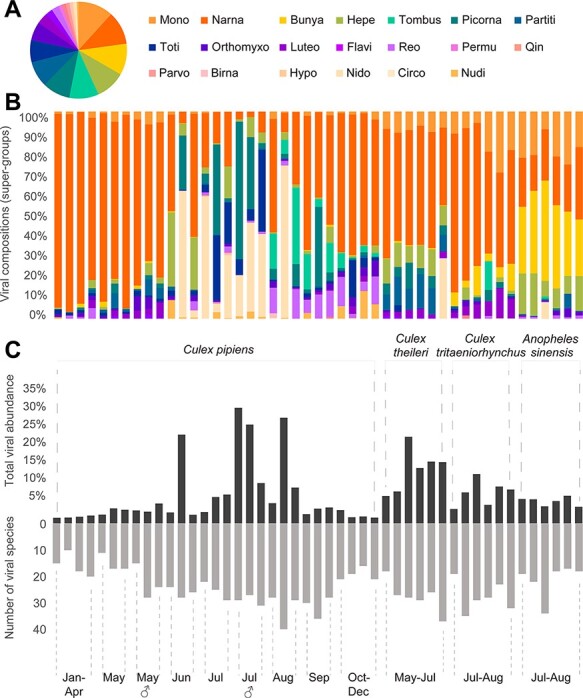
Overview of the mosquito eukaryotic virome determined in this study. (A) Proportion of eukaryotic virus species in each of the clades (RNA viruses) or families (DNA viruses). (B) Viral compositions of each pool. Time, host, and gender information for each pool follows Fig. 2C, and the viral diversity color scheme follows Fig. 2A. (C) Distribution of total viral abundance (top) and the number of viral species in the forty-eight pools. Time information is shown at the bottom of each bar, while host classifications are shown above the graph.

Meta-transcriptomic sequencing of forty-eight pools resulted in an average of 75,781,758 (range 59,410,848–95,817,340) reads per pool, assembled *de novo* into 24,164–410,121 contigs for virus discovery and characterization. These data revealed a huge diversity of eukaryotic viruses, comprising 162 species from seventeen major RNA virus supergroups (Shi et al. 2016) and three DNA virus families (*Circoviridae, Nudiviridae*, and *Parvoviridae*) (Fig. 2A, Table S1). Importantly, diversity compositions were highly variable across months, gender, and mosquito species. For example, the ‘Bunya-Arena’ clade was highly abundant in *Anopheles* mosquito pools but not in *Culex* pools. Similarly, the ‘Nido’ clade only appeared at high abundance in the summer months (Fig. 2B). Conversely, the ‘Narna-levi’ clade was consistent in all seasons and mosquito hosts and was commonplace (>10 per cent of total reads) in most pools (Fig. 2B).

In addition to diversity, we examined the abundance of the eukaryotic viruses discovered, which comprised 1.55–32.01 per cent of total non-rRNA reads in each pool, with an average of 8.2 per cent (Fig. 2C). The lowest abundance (1.55–2.16 per cent) was observed in *C. pipiens* mosquitoes during the colder months (i.e. January–April and October–December), whereas the highest abundance (>20 per cent of total RNA) was observed in the same mosquito species during summer months (i.e. June–August). There was no apparent correlation between viral diversity (i.e. number of species) and abundance based on these data (Fig. 2C).

### 3.3 Diversity of mosquito viruses

**Figure 3.**
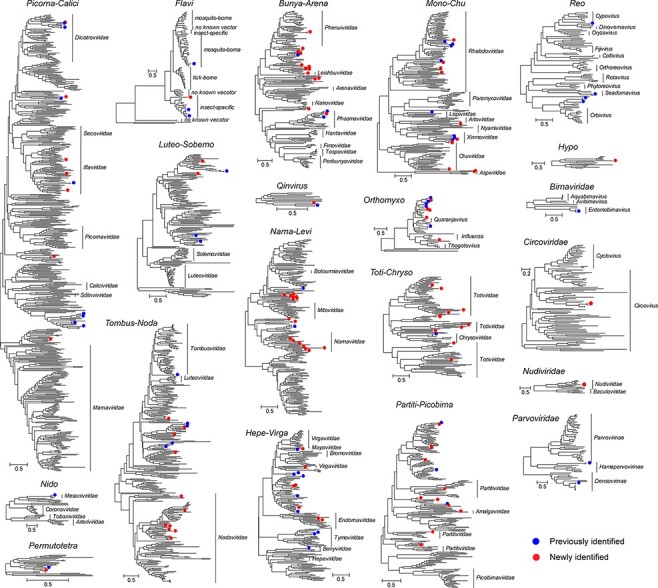
Genetic diversity of the viruses found in this study. A total of twenty phylogenetic trees are shown: seventeen based on RdRp alignments of major RNA virus clades (Shi et al. 2016) and three based on conserved replicase or nonstructural proteins of DNA viruses. Within each phylogeny, existing virus species are denoted by solid blue circles, while new viruses identified here are denoted by solid red circles. The names of the families or genera within each clade are shown to the right of each phylogeny. Each scale bar indicates 0.5 amino acid substitution per site.

A total of 162 virus species were identified here, among which 101 were assigned as potentially new species based on an amino acid identity threshold of 90 per cent in the RdRp (RNA viruses) or replication/nonstructural protein (DNA viruses) in comparison to their closest relatives. These viruses belonged to seventeen RNA virus super-clades (Shi et al. 2016) and three DNA virus families (Fig. 3). In the case of RNA viruses, forty-eight species of negative-sense, seventy-eight positive-sense, and thirty-two double-stranded viruses were identified, with the largest diversity was found in the Mono-Chu (*n* = 19), Narna-Levi (*n* = 18), and Bunya-Arena (*n* = 17) clades (Fig. 3, Figs S1–S7).

Our phylogenetic analysis revealed that the majority (*n* = 93) of the RNA viruses clustered with virus groups associated with mosquitoes or other insects. For example, we identified four members of the genus *Flavivirus*, one of which (JEV) belonged to the ‘mosquito-borne’ group, while the other three clustered in the ‘insect-specific’ group. The latter included a new species (XiangYun flavivirus) that was genetically distinct (51.17 per cent amino acid identity at the NS5 region) from existing members of the ‘insect-specific’ group (Fig. S3). Conversely, the DNA viruses identified in this study belonged to the *Circoviridae* (*n* = 1), *Nudiviridae* (*n* = 1), and *Parvoviridae* (*n* = 2, one within the *Hamaparvovirinae* and the other from the *Densovirinae*) (Fig. S7). The nudivirus identified here was a new species relatively close to Oryctes rhinoceros nudivirus (39.31 per cent identity) identified from *Oryctes rhinoceros*. RNA sequencing recovered ∼23.3 per cent of the viral genome, covering a set of well-characterized genes, including the DNA polymerase B protein, pif-1, GrBNV gp23-like protein, GrBNV gp97-like protein, late expression factor 4, DNA helicase, 19 kDa protein, and GrBNV gp13-like protein genes.

### 3.4 Virome diversity and evolution by host species, time, and gender

**Figure 4.**
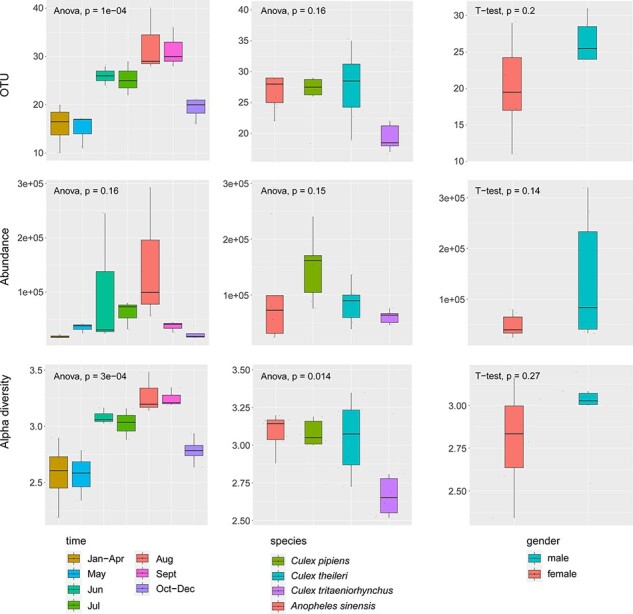
Comparisons of observed richness (OTU, upper row), abundance (RPM, middle row), and alpha diversity (lower row) between different months (left column), mosquito species (middle column), and gender (right column). An ANOVA test was performed for the time and species groups, while a *t*-test was used to text for associations with gender. *P* values are shown at the top left of each graph. In the boxplots, bold lines show the median, and upper and lower hinges show the first and third quartiles. Colors correspond to different ecological groups.

We first compared the observed richness, total viral abundance, and alpha diversity across mosquito species (i.e. *C. pipiens, C. theileri, C. tritaeniorhynchus*, and *A. sinensis*), different sampling months (January–December), and gender (male versus female). Strikingly, observed richness (operational taxonomic units (OTU), ANOVA test, *P* = 1e − 04) and alpha diversity (ANOVA test, *P* = 3e − 04) differed significantly across different times of year based on comparisons of female *C. pipiens* libraries across seven time periods (i.e. January–April, May, June, July, August, September, and October–December) (Fig. 4, left column). Overall, observed richness and alpha diversity were higher in the warmer months (i.e. from June to September) than the colder months (i.e. January–May or October–December) (Fig. 4, left column). Although virus abundance did not differ across all time points (ANOVA test, *P* = 0.16), pairwise comparisons between individual time periods revealed significantly higher abundance level in June, July, and August compared to January–April and October–December (*t*-tests, *P* values < 0.05).

With respect to comparisons between mosquito species, which were limited to female mosquitoes collected between May and August, we revealed substantial differences in alpha diversity (ANOVA test, *P* = 0.014) across the four species (Fig. 4, middle column). Pairwise comparisons indicated that the difference mainly existed between the *Culex* and *Anopheles* genera (*t*-tests, *P* values < 0.005), with a generally higher alpha diversity in *Culex* mosquitoes. However, no significant differences were found in observed richness or abundance comparisons across the four mosquito species (OTU, ANOVA test, *P* = 0.16; abundance, ANOVA, *P* = 0.15). Similarly, no significant differences were found in observed richness, abundance, or alpha diversity in comparisons of the mosquito (*C. pipiens*, May–July) virome between genders (OTU, *t*-test, *P* = 0.2; abundance, *t*-test, *P* = 0.14; alpha diversity, *t*-test, *P* = 0.27). Finally, a generalized linear model that included all three factors, namely, time, species, and gender, also revealed that alpha diversity was predicted by time and mosquito species, but not sex (Table S2), which is consistent with the results from separate comparisons.

**Figure 5.**
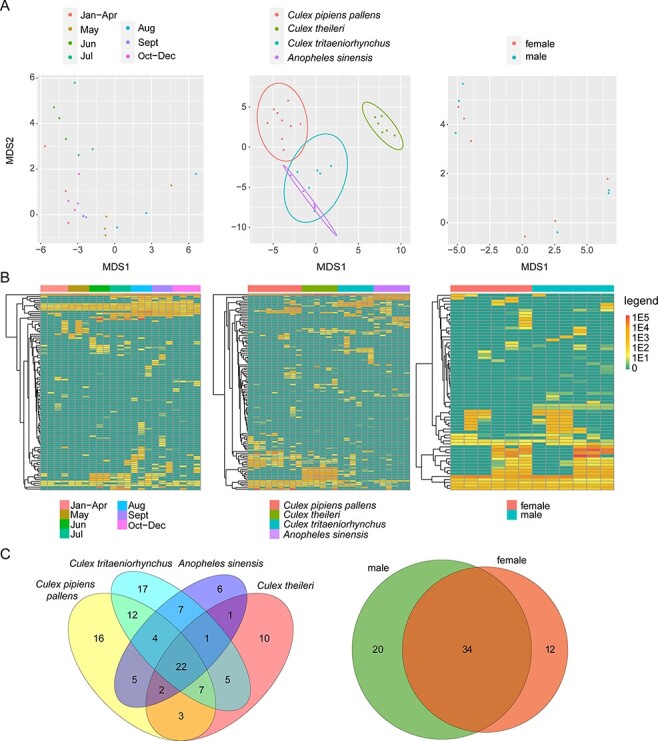
Effect of species, time, and gender on the mosquito virus community composition. (A) Multidimensional scaling plot (applying the Euclidean distance matrix) for viral composition over time, by species and by gender. The circles show the 95 per cent normal probability ellipse for each species group (middle panel). (B) Heatmap showing the normalized abundance of each viral species at different times, species, and gender groups. (C) Venn diagrams of the virus species shared between different hosts and gender. The size of the oval is not indicative of the number of viruses. Colors reflect different ecology groups.

We next performed multidimensional scaling to examine the effect of time, species, and gender on the mosquito virus community composition. This revealed that viral communities were primarily structured by mosquito species and less so by month/season or gender (Fig. 5A). Nevertheless, some monthly structuring was observed (Fig. 5A, left panel). Specifically, virome compositions in June and July were distinct from the remaining months, most likely due to increasing virus diversity and abundance (Fig. 5B). However, there was also substantial overlap among viromes (Fig. 5B). Of 118 virus species identified, sixty-nine (58.5 per cent) viruses were identified in more than two mosquito species and twenty-two (18.6 per cent) viruses were found in all four species (Fig. 5C). Similarly, thirty-four out of sixty-six virus species were shared between male and female mosquitoes from the same species and regions, suggesting substantial overlap of virome between genders.

Since these comparisons utilized total viromes, some of the viruses identified here may be associated with diet, parasites, or microbes within the mosquitoes rather than replicating in the mosquitoes themselves. To exclude the impact of these non-mosquito viruses, we identified and selected those that were most likely associated with true mosquito replication using three criteria: (i) they have been shown to infect mosquitoes or mosquito cells in previous studies; (ii) they clustered with other mosquito- or insect-related viruses on phylogenetic trees; and (iii) their abundance measured as RPM was greater than 1,000. Analyses repeated on this mosquito virus data subset generated similar results to those of the initial dataset (Fig. S8), suggesting that these observations are robust.

### 3.5 High-resolution characterization of individual virus species

**Figure 6.**
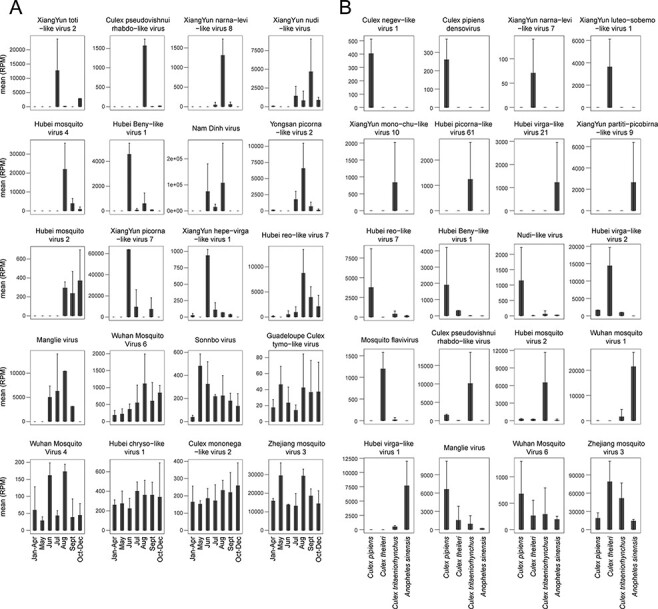
Temporal and host species distribution of selected individual viral species reflected by mean and standard deviation of abundance levels. (A) Temporal distribution of representative individual virus species. The viruses were selected and ordered based on their unique temporal distributions. Date information is provided at the bottom of each bar. The error bar represents standard deviation of mean. (B) Mosquito species distribution of representative individual virus species. The viruses were selected and ordered based on unique host distributions. Host information is shown at the bottom of each bar. The error bar represents standard deviation of mean.

For each individual viral species identified here we compared changes in prevalence and abundance over time. Two major temporal patterns were observed. A small number of viruses were characterized by a continuous presence and stable abundance all year round (e.g. Hubei chryso-like virus 1, Culex mononega-like virus 2, and Zhejiang mosquito virus 3). Interestingly, among those at stable abundance, Zhejiang mosquito virus 3 had much higher abundance (13,240–29,570 RPM) than Hubei chryso-like virus 1 and Culex mononega-like virus 2, which were present at 225–402 and 153–260 RPM, respectively (Fig. 6A). However, most viruses were not present all year round and some only appeared sporadically, although they may occasionally reach very high abundance (up to 107,768 RPM in the case of Nam Dinh Virus). Furthermore, seasonal variation is apparent in many virus species, with higher abundance and prevalence in months with higher temperatures (i.e. from May to September) than those with lower temperatures (i.e. January–April and October–December) (Fig. 6A).

We also investigated the host range for each of the virus species identified. This identified some generalist viruses that were present in all four mosquito species at high abundance (Fig. 6B), including Zhejiang mosquito virus 3 and Wuhan mosquito virus 6 which were also present throughout the year (Fig. 6A). The other virus species revealed strong host specificity or host preference. Specifically, Culex pipiens densovirus, XiangYun luteo-sobemo-like virus 1, and XiangYun partiti-picobirna-like virus 9 were only associated with *C. pipiens, C. theileri*, and *A. sinensis*, respectively. Conversely, although Hubei reo-like virus 7, Wuhan mosquito virus 1, and Hubei virga-like virus 1 were detected in multiple hosts, they were consistently at much higher prevalence, suggesting a strong host preference (Fig. 6B).

### 3.6 Characterization of vector-borne viruses

**Figure 7.**
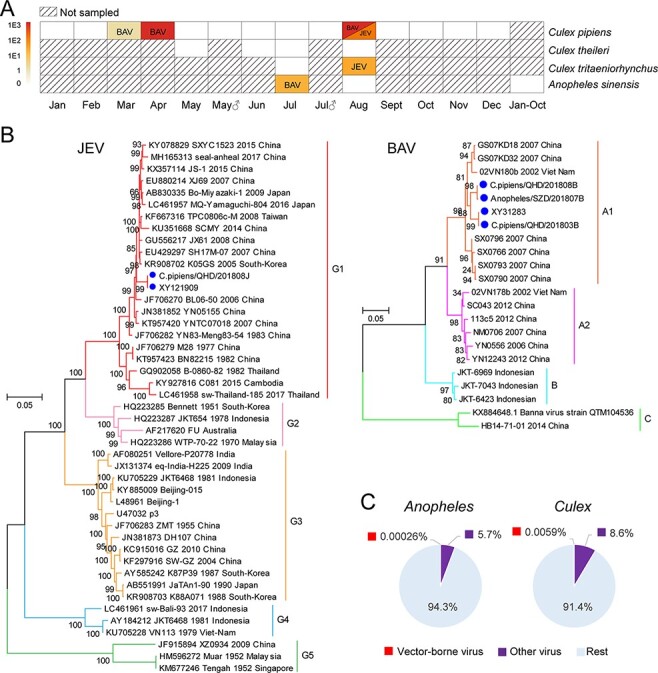
Discovery and characterization of vector-borne viruses. (A) The sampling time, mosquito host, and mosquito gender information of the pools positive for the vector-borne viruses JEV and BAV. Mosquito species information is shown on the right, while month and gender are shown at the bottom. The color of the box indicates both the positivity for vector-borne viruses and their corresponding abundance. The shaded box indicates that no sampling was performed in this category. (B) Genotyping of the JEV and BAV genomes sequenced identified in this study. The blue circle indicates vector-borne viruses identified in this study. Genotype information for JEV and BAV is shown to the right of each phylogeny. (C) Pie chart showing the proportion of total non-rRNA reads associated with vector-borne viruses and the entire virome for the *Culex* and *Anopheles* genera, respectively. Vector-borne viruses are marked in red and other viruses are marked in purple.

We also searched for viruses with a potential impact on human health, identifying those that were phylogenetically related to species or genera associated with vector-borne viruses, including flaviviruses, alphaviruses, bunyaviruses, and reoviruses. Two potential vector-borne viruses were identified: JEV and BAV, belonging to the genera *Flavivirus* and *Seadornavirus*, respectively (Fig. 7A). Among these, JEV was identified in one *C. pipiens* (Qinghua cave) and one *C. tritaeniorhynchus* pool (Shuizhangdi village), both sampled in August. In contrast, BAV was identified in March, April, and August from *C. pipiens* and in July from *A. sinensis* (Fig. 7A). Within each library, JEV had 26–155 RPM while BAV was at 1.5–1114 RPM abundance.

The JEV sequences identified in this study were named *C.pipiens*/QHD/201808J and XY121909 and were identical at the nucleotide level. Based on the phylogenetic analyses, they belonged to Genotype I and were closely related to variants from China, Japan, and Korea (Fig. 7B). The BAV sequences identified formed two different phylogenetic groups that shared 96.3 per cent genome sequence identity. Both groups belonged to the A1 genotype and were most closely related to sequences sampled in Vietnam (97.81–98.56 per cent, Fig. 7B).

Overall, our results indicate that vector-borne viruses represent only a tiny proportion of the total virome (Fig. 7C). Indeed, in the case of *Anopheles*, the proportion of total non-ribosomal RNA reads for vector-borne viruses was only 0.00026 per cent, some four orders of magnitude lower than that of the entire virome (5.7 per cent). With respect to *Culex*, the proportion was 0.0059 per cent for vector-borne viruses and 8.6 per cent for the entire virome.

## 4. Discussion

We performed a systematic time-series analysis of the mosquito virome, revealing its diversity and abundance over different mosquito species, sampling seasons, and genders. Although our analyses were limited to one location and four mosquito species, a highly diverse and abundant virome was documented, again demonstrating the capacity of arthropods to tolerate very high virus levels (Bose and Banerjee 2003; Li et al. 2015a; Shi et al. 2016; Wu et al. 2020). Indeed, this study alone identified 162 viral species from twenty families of DNA and RNA viruses. Among these, 101 viral species were novel, including the first mosquito-associated nudivirus (*Nudiviridae*, Fig. S7) as well as a new member of the genus *Flavivirus* (Fig. S3). Importantly, most of these newly identified viruses are likely to be mosquito viruses because they were related to those previously identified in mosquitoes, insects from the order Diptera, or other arthropod species and were often at high prevalence and/or abundance in mosquitoes and are hence unlikely to be associated with nonhost organisms. Hence, the presence of such high virus diversity in a small mosquito population again highlights that our understanding of mosquito virus diversity is far from complete (Atoni et al. 2018; Sadeghi et al. 2018; Shi et al. 2019; Du et al. 2020; Ramírez et al. 2020; Nebbak et al. 2021). In addition, the presence of arthropod-specific viruses in mosquitoes might impact the replication and transmission of arboviruses that also infect vertebrates, which has been demonstrated both *in vitro* (Kenney et al. 2014; Schultz, Frydman, and Connor 2018) and *in vivo* (Bolling et al. 2012). It will be of considerable interest to determine which, if any, of the arthropod-specific viruses discovered impacts arboviruses in this manner.

Meta-transcriptomics simultaneously reveals the diversity and abundance of the total virome, and it is now frequently used to explore viromes across different host species, geographical distributions, climate conditions, as well as sampling years (Wille et al. 2018, 2019, 2020; Du et al. 2020). Nevertheless, we believe this is the first characterization of a virome over the course of a single year. A key finding was the obvious seasonal pattern: the abundance and diversity of viruses were generally higher in summer than winter. Indeed, many of viruses discovered here, such as XiangYun picorna-like virus 7, Yongsan picorna-like virus 2, Hubei reo-like virus 7, as well as the vector-borne virus JEV, only appeared or had much higher abundance in warmer months. This is expected because seasonal variation in temperature, precipitation, and humidity contributes to the survival, replication, development, and distribution of mosquitoes, in turn impacting their virome structure (Bai, Morton, and Liu 2013; Li et al. 2018, 2019; Mordecai et al. 2020; Caldwell et al. 2021). For example, the reproductive activity of mosquitoes increases with temperature and rainfall, resulting in more efficient virus transmission (Tian et al. 2015). Other factors, such as mosquito population density and mammalian host activities, may also contribute to virome diversity and abundance, although this needs to be examined with more data.

While many viruses identified here experienced seasonal variation within the mosquito population, some were at stable presence throughout the year and characterized by 100 per cent pool prevalence rate and either high (i.e. Zhejiang mosquito virus 3, median 15,982 RPM) or moderate (i.e. Culex mononega-like virus 2, Hubei chryso-like virus 1, and Wuhan mosquito virus 4, median 45–342 RPM) abundance in *C. pipiens* (Fig. 6A). Among these, Culex mononega-like virus 2, Hubei chryso-like virus 1, and Zhejiang mosquito virus 3 viruses were also constantly detected in pooled *Culex australicus* and *Culex globocoxitus* sampled in Australia (Shi et al. 2017). Similarly, for individually sequenced mosquitoes, an almost ubiquitous presence (95.8–100 per cent prevalence) was observed for Wenzhou sobemo-like virus 4 and Wuhan mosquito virus 9 associated with *Culex* mosquitoes (Shi et al. 2019). The possible reasons why that these viruses are so commonplace include (i) a lack of adaptive immunity that is able to completely clear viral infection (Messaoudi et al. 2013; Gillich et al. 2015; Cheng et al. 2016, 2020; Goic et al. 2016; Filho et al. 2019; Lee et al. 2019; Reyes-Ruiz et al. 2019) and (ii) transovarial transmission such that viromes are passed from infected females to their progeny (Joshi, Mourya, and Sharma 2002; Lequime, Paul, and Lambrechts 2016; da Costa et al. 2017; Li et al. 2017; Yang 2017; Ferreira-de-lima and Lima-Camara 2018; Satoto et al. 2018; Heath et al. 2020). Unfortunately, however, due to limitations in winter sampling the hypothesis of over-wintering of these viruses cannot be addressed with the currently available data. Despite this, it is notable that most of the viruses identified do not persist over the course of the year. One possibility for this variation in prevalence is the fitness cost associated with virus infection. Indeed, it is possible that year-round presence is more often associated with viruses that have no or low fitness cost (Habayeb, Ekengren, and Hultmark 2006; Merkling and van Rij 2015; Wang et al. 2019), although this needs to be examined in the future studies.

Our study compared the viromes of four different mosquito species while controlling for location (i.e. Xiangyun county and Yunnan province) and season (i.e. summer) and revealed that overall viral compositions differ significantly across the four mosquito species—*C. pipiens, C. theileri, C. tritaeniorhynchus*, and *A. sinensis*. Previous studies have suggested that *Aedes aegypti* and *Culex quinquefasciatus* harbor very distinctive viromes (Marcelino et al. 2019), as do *Aedes* and *Culex* genera in general (Shi et al. 2017). Our study is also noteworthy because it identified barriers at the intra-genus level (i.e. between different *Cule*x species). Indeed, the majority of the viruses identified here showed either (i) host species specificity, such that they were only present in one species, or (ii) host species preference, such that they exhibited higher abundance in one particular species, although this is indistinguishable from a generalist virus causing out-of-phase epidemics in different species. Despite this, we did observe some generalist viruses, such as Wuhan mosquito virus 6 and Zhejiang mosquito virus 3 that appeared, at comparable abundance, in all four species (Fig. 6B). These viruses were also reported in Wuhan, China (*C. quinquefasciatus*) (Li et al. 2015a), and Australia (*C. australicus* and *C. globocoxitus*) (Shi et al. 2017), suggesting a much wider geographic distribution and host range for these viruses.

Although most of the viruses identified here belonged to the arthropod-specific category that can be distinguished from the vertebrate-infecting groups based on phylogenetic analyses, we identified two mosquito-borne viruses—JEV and BAV—that have been isolated in diseased humans as well as domestic animals in China (Gao, Nasci, and Liang 2010; Zheng et al. 2012). However, these arboviruses only appeared sporadically, with only a low pool prevalence and low to moderate abundance (Fig. 7). This accords with previous studies in which arboviruses only represent a small proportion of the total mosquito viral population (Li et al. 2015a; Shi et al. 2017; Du et al. 2020). Nevertheless, the presence of JEV in both locations investigated in this study, consistent with a previous report that isolated 17 JEV strains from Shuizhangdi village (Pan et al. 2020), suggests a potential risk of human exposure. Conversely, previous viral surveillance of bat serum collected from Qinghua cave did not find JEV but did identify Yokose virus (YOKV), a flavivirus isolated from a serum sample of *Myotis daubentonii* (Daubenton’s bat; Feng et al. 2019). However, YOKV was not identified in the mosquito samples here, compatible with its status a member of the ‘no known vector’ (i.e. NKV flavivirus) group (Blitvich and Firth 2017) such that mosquitoes may not be involved in its transmission. Alternatively, the virus may be transmitted by a day-time feeder from the genus *Aedes* which were not sampled in this study.

Despite its size, this study has several limitations. First, meta-transcriptomics does not provide data on the functional viability of newly discovered viruses such that future isolation of these viruses is needed to confirm their existence as transmissible viral particles. Second, only female *C. pipiens* were sampled across different months, whereas sampling of other mosquito species and genders were limited to the summer months, limiting our capability to study the interactions among different factors. Third, some potentially important data, such as mosquito population size, temperature, humidity, and anthropogenic activities, were not collected, limiting our capability to reveal all the factors that influence virome structure. Finally, although Qinghua cave and Shuizhang village were only 5 km apart, they have distinct ecological features. While it is difficult to distinguish their effect with current data, future studies are needed to understand the potential impact of geographical/ecological factors on the virome structure.

Collectively, the data generated here revealed strong seasonal variation and strong host structure among the mosquitoes sampled but no substantial differences by gender. Notably, some viruses displayed a ubiquitous presence throughout the year and across four different mosquito species, although the majority was season and host specific. More broadly, our study further highlights how meta-transcriptomics can be used to reveal the diversity of the mosquito virome at both great depth and precision.

## Supplementary Material

veac006_Supp

## Data Availability

All sequencing reads have been deposited in the SRA databases under the project accession PRJNA778885. Relevant virus genome/gene sequences have been deposited in GenBank under the accessions OL700045–OL700212.
