# Supplementary material for: A time-series meta-transcriptomic analysis reveals the seasonal, host, and gender structure of mosquito viromes
Source: Virus Evol. 2022 Feb 2;8(1):veac006. doi: 10.1093/ve/veac006 (PMC8887699; doi:10.1093/ve/veac006)
Supplement: veac006_Supp [file veac006_supp.zip › supplementary figure.pdf]

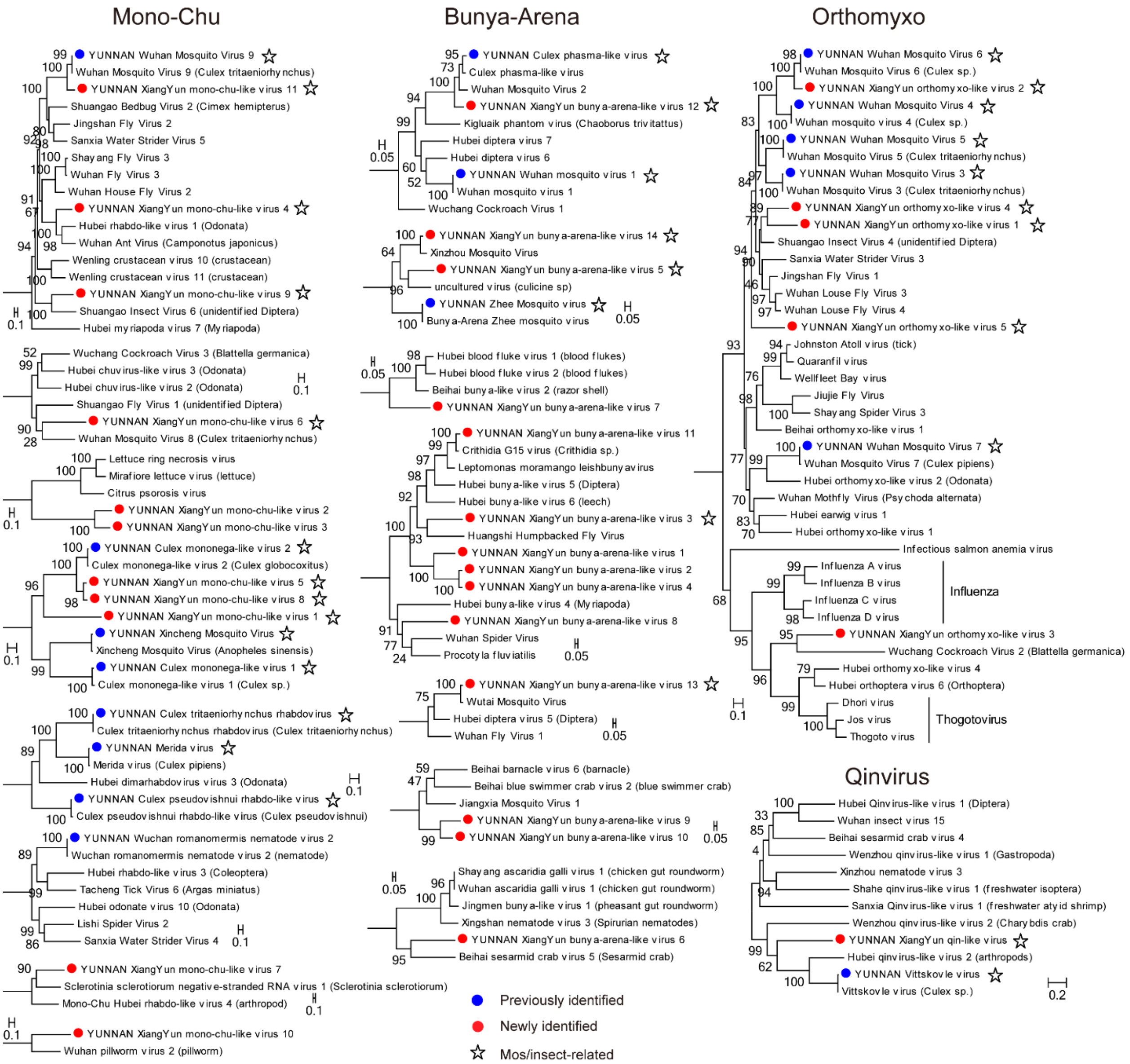

Figure S1. Phylogenetic trees of the viruses identified in the Mono-Chu, Bunya-Arena, Orthomyxo and Qinvirus groups of RNA viruses. Host information (in parentheses) is provided after the names of the reference viruses. The majority of these viruses clustered with mosquito- or insect-associated virus groups (denoted by stars). Within each tree, the viruses identified previously are denoted by solid blue circles, while those identified in this study are denoted by solid red circles. Reference viruses are unmarked. The tree is midpoint rooted for clarity only.

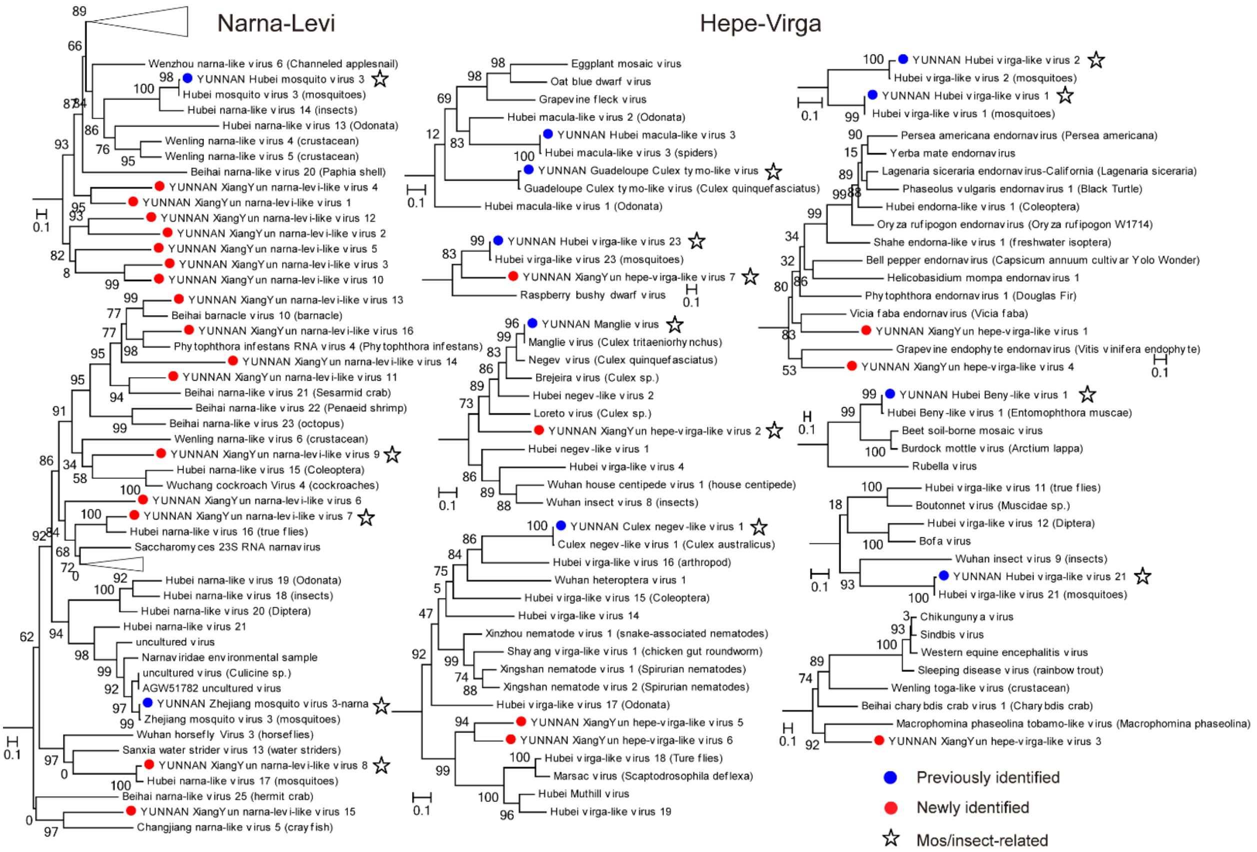

Figure S2. Phylogenetic trees of the viruses identified in the Narna-Levi and Hepe-Virga groups of RNA viruses. Host information (in parentheses) is provided after the names of the reference viruses. The majority of these viruses clustered with mosquito- or insect-associated virus groups (denoted by stars). Within each tree, the viruses identified previously are denoted by solid blue circles, while those identified in this study are denoted by solid red circles. Reference viruses are unmarked. The tree is midpoint rooted for clarity only.

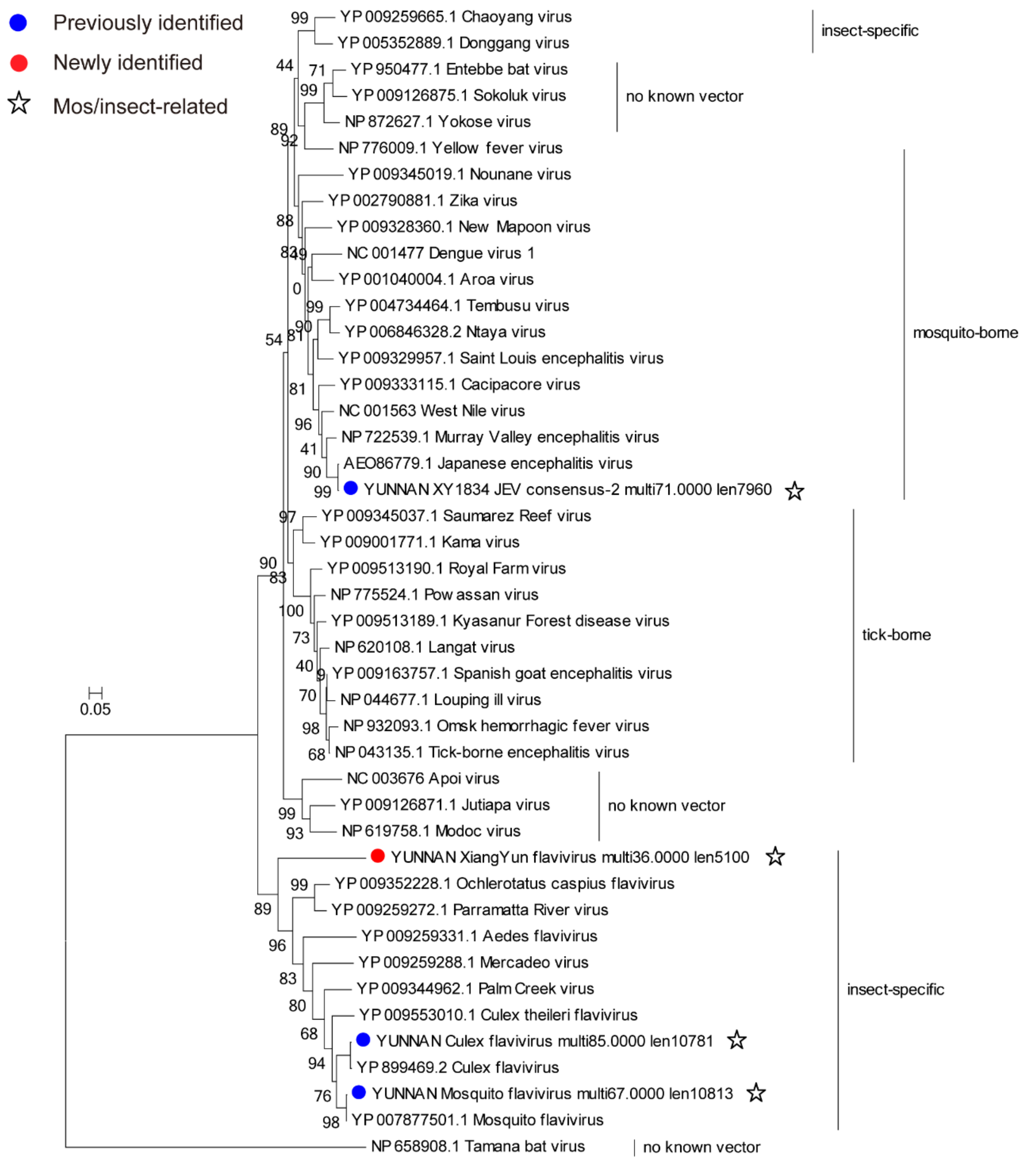

Figure S3. Phylogenetic trees of the viruses identified in the "Flavi" group of RNA viruses. Host information (in parentheses) is provided after the names of the reference viruses. The majority of these viruses clustered with mosquito- or insect-associated virus groups (denoted by stars). Within each tree, the viruses identified previously are denoted by solid blue circles, while those identified in this study are denoted by solid red circles. Reference viruses are unmarked. The tree is midpoint rooted for clarity only.

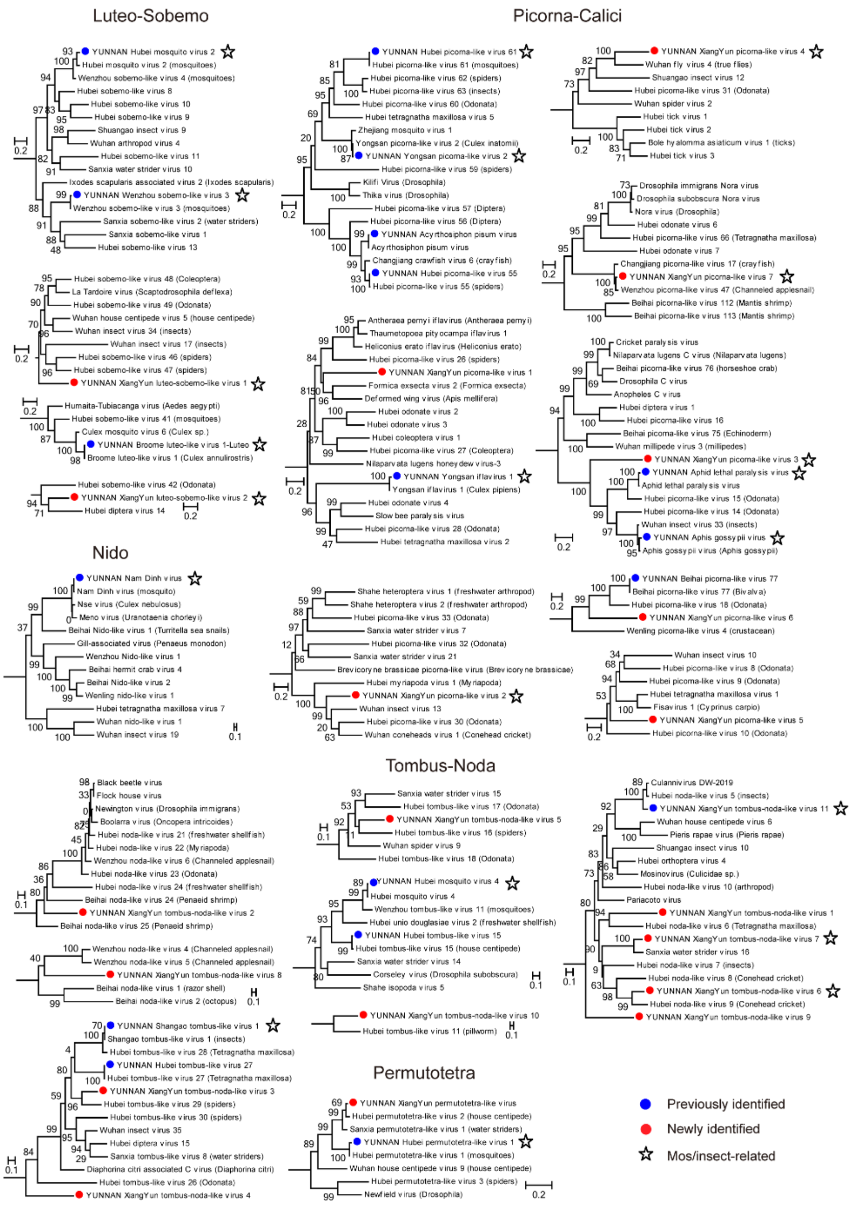

Figure S4. Phylogenetic trees of the viruses identified in the Tombus-Noda, Picorna-Calici, Luteo-Sobemo, Nido and Permutotetra groups of RNA viruses. Host information (in parentheses) is provided after the names of the reference viruses. The majority of these viruses clustered with mosquito- or insect-associated virus groups (denoted by stars). Within each tree, the viruses identified previously are denoted by solid blue circles, while those identified in this study are denoted by solid red circles. Reference viruses are unmarked. The tree is midpoint rooted for clarity only.

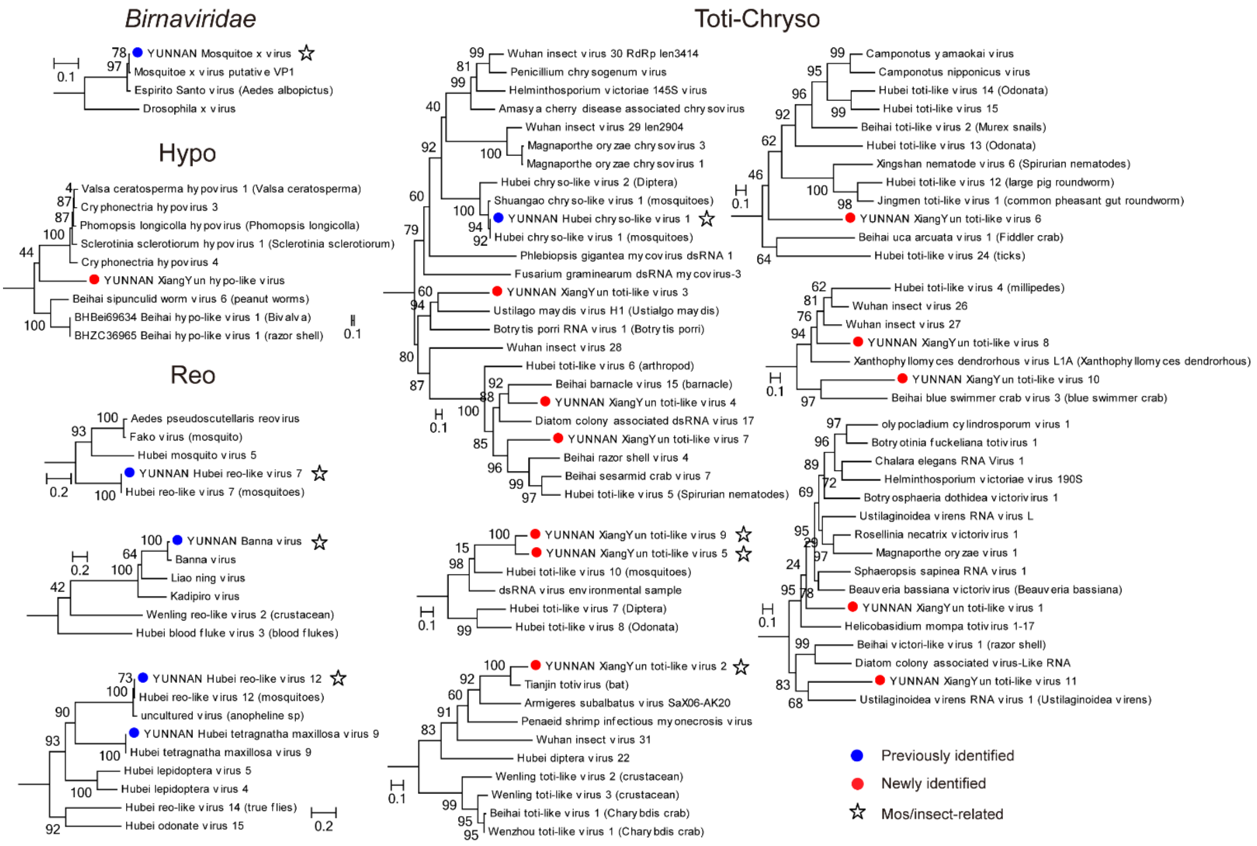

Figure S5. Phylogenetic trees of the virus identified in the Birnaviridae, Hypo, Reo and Toti-Chryso groups of RNA viruses. Host information (in parentheses) is provided after the names of the reference viruses. The majority of these viruses clustered with mosquito- or insect-associated virus groups (denoted by stars). Within each tree, the viruses identified previously are denoted by solid blue circles, while those identified in this study are denoted by solid red circles. Reference viruses are unmarked. The tree is midpoint rooted for clarity only.

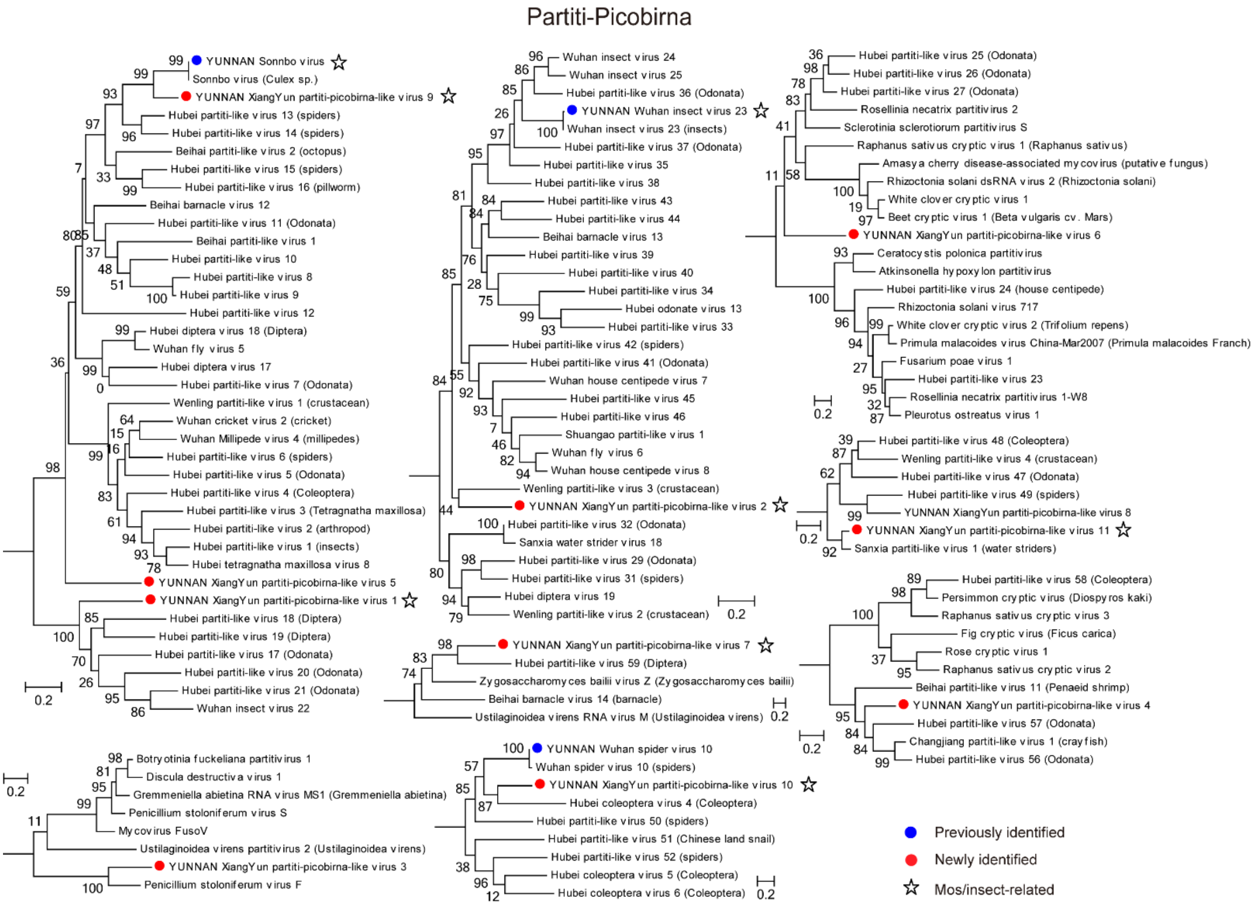

Figure S6. Phylogenetic trees of the virus identified in the Partiti-Picobirna group of RNA viruses. Host information (in parentheses) is provided after the names of the reference viruses. The majority of these viruses clustered with mosquito- or insect-associated virus groups (denoted by stars). Within each tree, the viruses identified previously are denoted by solid blue circles, while those identified in this study are denoted by solid red circles. Reference viruses are unmarked. The tree is midpoint rooted for clarity only.

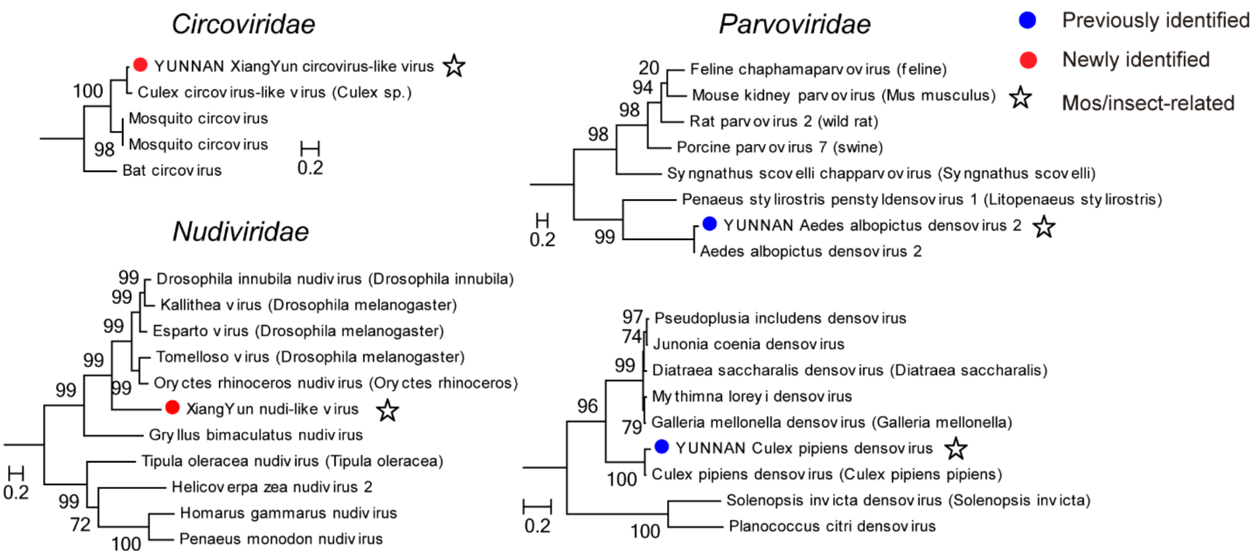

Figure S7. Phylogenetic trees of the viruses identified in the DNA virus families *Parvoviridae*, *Circoviridae* and *Nudiviridae*. Host information (in parentheses) is provided after the names of the reference viruses. The majority of these viruses clustered with mosquito- or insect-associated virus groups (denoted by stars). Within each tree, the viruses identified previously are denoted by solid blue circles, while those identified in this study are denoted by solid red circles. Reference viruses are unmarked. The tree is midpoint rooted for clarity only.

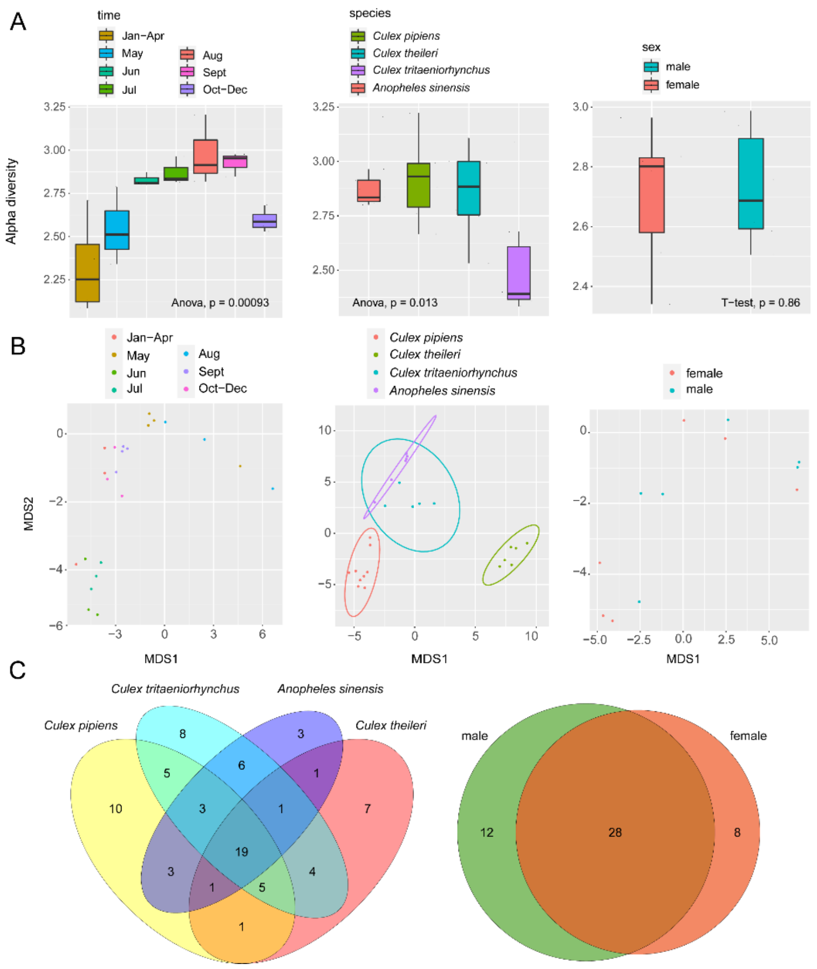

Figure S8. Virome composition of mosquito/insect-related viruses across ecological groups. (A) Comparisons of Alpha diversity between different months (left), mosquito species (middle), and gender (right). An ANOVA test was used for time and species groups, while a t-test was used for gender groups. P-values are shown at the top left of each graph. In the boxplots, bold lines show the median, and upper and lower hinges show the first and third quartiles. Colors correspond to different ecological groups. (B) Multidimensional scaling plot (Euclidean distance matrix) for viral composition compared over time, species and gender. The circles show the 95% normal probability ellipse for each species group (middle panel). (C) Venn diagrams showing virus species shared between different hosts and gender. The size of the oval is not indicative of the number of viruses. Colors correspond to different ecological groups.
