# Supplementary material for: A time-series meta-transcriptomic analysis reveals the seasonal, host, and gender structure of mosquito viromes
Source: Virus Evol. 2022 Feb 2;8(1):veac006. doi: 10.1093/ve/veac006 (PMC8887699; doi:10.1093/ve/veac006)
Supplement: veac006_Supp [file veac006_supp.zip › Table S2.docx]

Table S2 Generalized Linear Model of Alpha diversity over time, gender and species

| Groups | Df | Deviance | Resid. Df | Resid. Dev | Pr(>Chi) | Signif. |
| --- | --- | --- | --- | --- | --- | --- |
| NULL |  |  | 46 | 3.8777 |  |  |
| time | 6 | 1.19126 | 40 | 2.6865 | 4.384e-05 | *** |
| gender | 1 | 0.03191 | 39 | 2.6546 | 0.371995 |  |
| species | 3 | 0.51481 | 36 | 2.1397 | 0.004956 | ** |
| time:gender | 1 | 0.60658 | 35 | 1.5332 | 9.937e-05 | *** |
| time:species | 4 | 0.29187 | 31 | 1.2413 | 0.121380 |  |
| gender:species | 0 | 0.00000 | 31 | 1.2413 |  |  |
| time:gender:species | 0 | 0.00000 | 31 | 1.2413 |  |  |

Signif. codes: 0 ‘***’ 0.001 ‘**’ 0.01 ‘*’ 0.05 ‘.’ 0.1 ‘ ’ 1
